# An Ancient Boxing Exercise Improves Physical Functions, Balance, and Quality of Life in Healthy Elderly Persons

**DOI:** 10.1155/2018/6594730

**Published:** 2018-12-03

**Authors:** Taweesak Janyacharoen, Thitipa Srisamai, Kittisak Sawanyawisuth

**Affiliations:** ^1^School of Physiotherapy, Faculty of Associated Medical Sciences, Khon Kaen University, Khon Kaen, Thailand; ^2^Research Center in Back, Neck, Other Joint Pain and Human Performance (BNOJPH), Khon Kaen University, Khon Kaen, Thailand; ^3^Department of Internal Medicine, Faculty of Medicine, Khon Kaen University, Khon Kaen, Thailand

## Abstract

It has been shown that traditional dances can be effective in improving physical functions in the elderly persons. Unlike other traditional dance exercises, the ancient Thai boxing exercise may be suitable for elderly persons in other ethnicities who are interested in boxing. This randomized controlled study aimed to evaluate the effects of the exercise on physical functions in elderly subjects. Healthy elderly subjects were recruited and randomly divided into two groups: the control group and the ancient Thai boxing group. The control group received education about the exercise and a home program of daily practice. The ancient Thai boxing group performed the modified ancient Thai boxing exercise for 12 weeks. There were six outcomes in this study which were recorded at baseline and at the end of study (week 12) including a six-minute walk test (6MWT), five times sit to stand test (FTSST), flexibility by trunk flexometer, time up and go test (TUGT), and Berg balance scale (BBS), as well as a test to determine quality of life (QOL). All outcomes were compared to the baseline, as well as between groups. There were 56 subjects enrolled in the study, 28 in the control group, and 28 in the ancient Thai boxing group, with mean ages of 68.6 and 65.9 years, respectively. The majority of subjects in both groups were women (96.4% and 89.3%). After 12 weeks of study, significant differences were found in terms of all seven outcomes between the two groups. For example, the 6MWT in the control group was 415.8 vs 480.3 m in the ancient boxing group. In conclusion, the 12-week ancient boxing exercise significantly improved physical functions, balance, and QOL in the elderly.

## 1. Introduction

The elderly make up a significant portion of the population in various countries around the world. Increased age can lead to cardiovascular changes and diseases such as myocardial infarction or stroke [1, 2]. In elderly patients with cardiovascular diseases response to treatment may be unpredictable or unfavorable [1]. The American patients aged over 65 years accounted for 82% of the 445,687 deaths from coronary heart disease in 2005 [2]. In addition, the cost for cardiovascular diseases is expected to be $475 billion and results mostly from the elderly patients [3, 4].

Physical function is an important factor associated with future cardiovascular diseases. Regular exercise has been clearly shown to improve physical functions [5]. A study found that obese and inactive elderly women had significantly lower physical function significantly from others even obese/active ones (182.5 vs 191.5). Dance has been shown to possibly improve physical function in the elderly subjects [6, 7]. In a previous study, scores on a six-minute walk test significantly improved subjects with a mean age of 73.5 years practiced a Turkish dance (419.1 to 488.8 m) [6]. An ancient Thai boxing exercise is one of Thai traditional dances which is moderate intensity exercise. Due to the slow rhythm, it may be suitable for the elderly people. There has been no previous study conducted to evaluate the effects of the ancient Thai boxing exercise on physical functions in the elderly. This randomized controlled trial, therefore, aimed to assess the effects of this exercise in elderly subjects.

## 2. Materials and Methods

This was an open labelled, randomized controlled trial conducted at Thailand's Sakon Nakhon province. Subjects were recruited from a local community center. The inclusion criteria were that they were 60 years old, able to walk at least 10 meters without assistance, and had not engaged in regular exercise within the past two months. Subjects were excluded if one of the following conditions was present: neurological impairment, Mini-Mental State Examination (Thai version) score less than 24, severe cardiovascular disease, persistent joint pain, musculoskeletal impairment, receipt of medication that affected balance, or severe pain (pain scale greater than five) that could affect the exercise. The study protocol was approved by the Khon Kaen University ethic committee in human research (HE592199).

All eligible subjects were randomly divided by simple random sampling into two groups: the control group and exercise group. The control group received education with regard to the exercise and home program to aid in daily practice. The ancient boxing group performed the exercise for 12 weeks. The exercise program was scheduled three times/week as a group exercise led by a trained instructor. Each session was comprised of five minutes of warm-up, 30 minutes of dance, and five minutes of cool-down, for a total of 40 minutes per session.

The exercise that was practiced is a type of native Thai boxing dance originating from Sakon Nakhon province in northeast Thailand (called* Phuthai Noi*). This exercise is an ancient folk dance accompanied by rhythmic drums and singing. The type of boxing on which it is based consists of 14 postures into which arms and legs are gently, slowly, and continuously positioned. In this study, only 12 postures were used in the 30-minute period, as the other two postures were not appropriate for the elderly to perform. All 12 postures are shown in the appendix a video posted online at https://goo.gl/ZAdhoU.

All subjects' baseline characteristics and outcomes were measured. There were six outcomes in this study that were recorded both at baseline and the end of study (week 12) including six-minute walk test (6MWT), five times sit to stand test (FTSST), flexibility by trunk flexometer, time up and go test (TUGT), Berg balance scale (BBS), and score on the World Health Organization Quality of Life-BREF-Thai version (QOL). The first five outcomes were measured using the standard protocol detailed in previously published articles [6, 8, 9]. The QOL comprised four domains including physical, psychological, social relationship, and environment aspect. Each domain had a score of 25 and sums up as a total score of 100 indicating the best QOL. Each outcome indicated physical functions as follows: 6MWT: endurance; FTSST: leg strength; flexibility: trunk flexibility; TUGT and BBS: balance.

### 2.1. Sample Size Calculation

There have been no previous studies of elderly subjects practicing this exercise over 12 weeks. We used a previous study of Thai dance in the elderly to calculate the sample size [6], using the 6MWT as the primary outcome of the study. The 12-weeks of Thai dance gave the effect size of 33.79 meters with a standard deviation of 39.38. In order to attain 80% power and 95% confidence, the required sample size was found to be 22 subjects/group. After adjusting for a 25% dropout rate, the sample size for each group was determined to be 28 subjects.

### 2.2. Statistical Analysis

Descriptive statistics were used to execute the baseline characteristics of participants in both groups. If the outcomes were normally distributed, the differences between baseline data and outcomes were compared by using a paired t test. The differences in outcome variables between the two groups at the end of the study were compared by using an independent t test. The level of significance was declared there was a *p* value < .05. All statistical analysis was executed using SPSS (version 17), Chicago, Illinois, USA.

## 3. Results

There were 56 subjects participated in the study: 28 in the control group and 28 in the ancient boxing group, with mean ages of 68.6 and 65.9 years, respectively. The majority of subjects in both groups were women (96.4% and 89.3%), as shown in Table 1. Other baseline variables were comparable between the two groups (Table 1).

After 12 weeks of study, all seven outcomes in both groups differed significantly from the baseline values (Table 2). All seven outcome variables also differed significantly between the two groups at the end of the study (Table 2). For example, the control 6MWT score in the control group at week 12 was significantly lower than at baseline (415.8 vs 449.5 m). A similar change was seen in the ancient boxing group (480.3 vs 462.2 m). At week 12^th^, the 6MWT scores differed significantly between the two groups (415.8 vs 480.3 m).

At the end of the study, the control group showed a statistically significant increase in TUGT scores (8.2 to 8.7 sec), whereas those of the ancient boxing group had significantly decreased (9.0 to 7.5 sec). In addition, BBS had significantly decreased in the control group (53.3 to 51.2), whereas it had statistically increased significantly in the ancient boxing group (52.1 to 54.9). The control group also showed a statistically significant difference in terms of TUGT score and BBS when compared with experimental group (Table 2).

The control group exhibited a statistically significant decrease in QoL (90.0 to 87.1), whereas QoL had significantly increased in the ancient boxing group from 89.8 to 95.9 (Table 3). In addition, the control group exhibited a statistically significant decrease in the physical (22.7 to 21.2) and psychological (23.0 to 21.5) domain whereas these had significantly increased in the ancient boxing group (22.3 to 25.6 and 22.7 to 24.8). There was also a statistically significant difference in QoL between the control and experimental group in these two domains (Table 3).

## 4. Discussion

The present study showed that the 12-week ancient boxing exercise program resulted in significant improvement in all outcomes compared with the control group, as well as from baseline (Table 2). As previously reported, aerobic exercise including dance has been shown to improve physical functions in the elderly [10, 11].

Compared with other traditional dances, engagement in this ancient boxing exercise resulted in significant improvement in physical functions, flexibility, balance, and QOL. Previous studies examining Turkish and Thai dance in elderly participants found improvement in only some outcomes [6, 7]. A Turkish folk dance was found to improve 6MWT by 69.7 meters and BBS by 1.2 units, while the Thai dance improved 6MWT by 56.6 meters and flexibility by 4.5 cm, in addition to reducing FTTS by 2.7 sec. The ancient boxing exercise in this study resulted in somewhat lower improvement levels of improvement compared with the other two exercises. In this study, subjects' 6MWT scores or endurance improved by 18.1 meters and flexibility improved by 1.3 cm (Table 2). Note that BBS improvement was slightly higher than that from the Turkish dance (2.8 vs 1.2) [7]. These findings may be explained by the baseline characteristics of the participants. The participants in this study appeared to be healthier or more physically active than those in the other two studies [6, 7]. For example, the baseline 6MWT and flexibility scores in this study were much higher than those in the other two studies (6MWT = 462.2 vs 419.1 vs 360.1 meters; flexibility = 15.0 vs 10.4 cm). This may have caused the percentage of improvement to be lower than in the other two studies.

An additional benefit from the ancient boxing exercise was improvement in quality of life. Participants' overall QOL was significantly better after 12 weeks of ancient boxing exercise (89.8 to 95.9), as shown in Table 2. The QOL was better after the improvement of all measured physical outcomes in this study resulting in significant improvement in both physical and phychological domain of QOL (Table 3). The Turkish folk dance mentioned above also improved QOL by an SF-36 form after an eight-week intervention [7]. However, only three out of eight domains had improved including physical functioning (79.1 to 88.8), mental health (69.3 to 81.0), and general health (63.0 to 65.1), similar to the QOL improvements found in in this study (Table 3). The advantages of this study were randomized controlled trial fashion and characters of exercise. Unlike other traditional dance exercises, the ancient boxing exercise may be suitable for elderly persons in other ethnicities who are interested in boxing. Finally, all participants enjoyed the exercise and were able to complete the 12-week study program.

There were some limitations to this study. First, over 90% of participants were women. The results may not be applicable to male participants. Second, the measured outcomes may represent or relate to other outcomes, such as flexibility indicating arterial stiffness, TUGT results indicating risk of fall or resting blood pressure, or heart rate indicating cardiovascular outcomes, which were not evaluated in this study. Third, some outcomes in the control group may have worsened from baseline. However, this result may not be clinically significant. Finally, all participants in the study were quite healthy. Thus, the results of this study may not be applicable to subjects with disability. BBS may not be the suitable indicator for balance in this study due to ceiling effect and high baseline values (52-53/56). Sharp Romberg test or sharp SLS may be more suitable measurement in healthy elderly subjects.

In conclusion, the 12 weeks of ancient boxing exercise significantly improved physical functions, balance, and QOL in the elderly.

## Figures and Tables

**Table 1:** Anthropometric and baseline characteristics of subjects in the control and ancient boxing group.

**Variables**	**Control group (n = 28)**	**Ancient boxing group (n = 28)**	***p*-value**
Gender (m/f)	1/27	3/25	0.30
Age (years)	68.6 ± 6.8	65.9 ± 4.9	0.09
Body weight (kg)	48.1 ± 7.8	44.1 ± 10.8	0.11
Height (cm)	151.0 ± 4.9	152.3 ± 8.9	0.48
SBP (mmHg)	125.5 ± 18.0	120.6 ± 18.0	0.30
DBP (mmHg)	68.6 ± 11.6	66.3 ± 10.4	0.42
HR (beat/minute)	82.8 ± 8.6	79.2 ± 9.8	0.14

Note: Values are mean ± SD, kg; kilograms, cm; centimeters, m; meter, SBP; systolic blood pressure, mmHg; millimeters of mercury, DBP; diastolic blood pressure, HR; heart rate.

**Table 2:** Physical performance and quality of life scores of subjects in the control and ancient boxing groups at week 0 and at the end of the study (n = 28) in each group.

**Variable**	**Groups**	**Week 0**	**Week **12^**th**^	***p*-value** ^**a**^	***p*-value** ^**b**^
6MWT (m)	Control	449.5 ± 25.7	415.8 ± 25.1^*∗*^	0.038	0.027
	Experimental	462.2 ± 48.5	480.3 ± 49.7^*∗*#^	0.031	

FTSST (sec)	Control	11.2 ± 2.6	12.2 ± 2.9^*∗*^	0.044	0.024
	Experimental	10.6 ± 3.0	9.3 ± 2.0^*∗*#^	0.036	

Flexibility (cm)	Control	12.2 ± 4.7	11.2 ± 4.6^*∗*^	0.046	0.019
	Experimental	15.0 ± 6.3	16.3 ± 6.3^*∗*#^	0.036	

TUGT (sec)	Control	8.2 ± 2.1	8.7 ± 2.1^*∗*^	0.042	0.029
	Experimental	9.0 ± 1.7	7.5 ± 1.1^*∗*#^	0.037	

BBS (scales)	Control	53.3 ± 1.8	51.2 ± 2.5^*∗*^	0.043	0.046
	Experimental	52.1 ± 5.1	54.9 ± 1.5^*∗*#^	0.042	

Note: Values are mean ± standard deviation; ^*∗*^significantly difference from corresponding at week 0 (*p* < 0.05); ^#^significantly difference from control group, 6MWT: six-minute walk test; FTSST: Five times sit to stand test; TUGT: Time up and go test; BBS: Berg balance scale; a: between week 0 and 12^th^; b: between groups.

**Table 3:** Quality of life scores in overall and by domains of subjects in the control and ancient boxing group groups at week 0 and at the end of the study (n = 28) in each group.

**Variables/domain**	**Groups**	**Week 0**	**Week **12^**th**^	***p*-value** ^**a**^	***p*-value** ^**b**^
Overall	Control group	90.0 ± 7.0	87.1 ± 5.7	0.028	0.011
	Ancient boxing group	89.8 ± 8.1	95.9 ± 8.3	0.017	

Physical domain	Control group	22.7 ± 3.0	21.2 ± 2.3	0.041	0.018
	Ancient boxing group	22.3 ± 2.4	25.6 ± 3.2	0.029	

Psychological domain	Control group	23.0 ± 2.5	21.5 ± 2.1	0.042	0.048
	Ancient boxing group	22.7 ± 1.9	24.8 ± 2.3	0.037	

Social Relationships	Control group	12.8 ± 0.9	12.7 ± 0.8	0.451	0.204
	Ancient boxing group	12.8 ± 0.9	13.0 ± 1.0	0.118	

Environment	Control group	25.2 ± 2.5	25.0 ± 2.2	0.346	0.307
	Ancient boxing group	25.7 ± 4.0	25.3 ± 3.8	0.241	

Note: Values are mean ± standard deviation; a: between week 0 and 12^th^; b: between groups.

## Data Availability

The data used to support the findings of this study are available from the corresponding author upon request.

## Supplementary Materials

Supplementary Material Details of 12 postures of ancient boxing exercise are described. A video of ancient boxing exercise is posted online at https://goo.gl/ZAdhoU.

## References

[1] Jackson C. F., Wenger N. K. (2011). Cardiovascular Disease in the Elderly. *Revista Española de Cardiología*.

[2] Yazdanyar A., Newman A. B. (2009). The burden of cardiovascular disease in the elderly: morbidity, mortality, and costs. *Clinics in Geriatric Medicine*.

[3] Lloyd-Jones D., Adams R., Carnethon M. (2009). Heart disease and stroke statistics--2009update: a report from the American Heart Association Statistics Committee and StrokeStatistics Subcommittee. *Circulation*.

[4] Hodgson T. A., Cohen A. J. (1999). Medical care expenditures for selected circulatory diseases: Opportunities for reducing national health expenditures. *Medical Care*.

[5] Brach J. S., VanSwearingen J. M., FitzGerald S. J., Storti K. L., Kriska A. M. (2004). The relationship among physical activity, obesity, and physical function in community-dwelling older women. *Preventive Medicine*.

[6] Janyacharoen T., Laophosri M., Kanpittaya J., Auvichayapat P., Sawanyawisuth K. (2013). Physical performance in recently aged adults after 6 weeks traditional Thai dance: A randomized controlled trial. *Clinical Interventions in Aging*.

[7] Eyigor S., Karapolat H., Durmaz B., Ibisoglu U., Cakir S. (2009). A randomized controlled trial of Turkish folklore dance on the physical performance, balance, depression and quality of life in older women. *Archives of Gerontology and Geriatrics*.

[8] Li K., Kay N. S., Nokkaew N. (2009). The performance of the world health organization's WHOQOL-BREF in assessing the quality of life of Thai college students. *Social Indicators Research*.

[9] Langley F. A., Mackintosh S. F. (2007). Functional Balance Assessment of Older Community Dwelling Adults: A Systematic Review of the Literature. *The Internet Journal of Allied Health Sciences and Practice*.

[10] Hui E., Chui B. T.-K., Woo J. (2009). Effects of dance on physical and psychological well-being in older persons. *Archives of Gerontology and Geriatrics*.

[11] Hollmann W., Strüder H. K., Tagarakis C. V. M., King G. (2007). Physical activity and the elderly. *European Journal of Preventive Cardiology*.

