# Supplementary material for: An Ancient Boxing Exercise Improves Physical Functions, Balance, and Quality of Life in Healthy Elderly Persons
Source: Evid Based Complement Alternat Med. 2018 Dec 3;2018:6594730. doi: 10.1155/2018/6594730 (PMC6304610; doi:10.1155/2018/6594730)
Supplement: Supplementary Material — Details of 12 postures of ancient boxing exercise are described. A video of ancient boxing exercise is posted online at https://goo.gl/ZAdhoU. [file 6594730.f1.docx]

**An ancient boxing exercise is** consisted of 12 postures and can be practiced with the Phu-Tai Noi music as follows:

1. **Swing arm position**


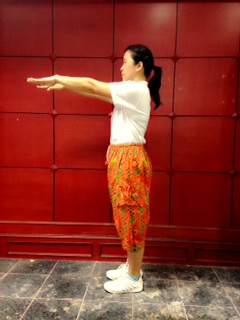

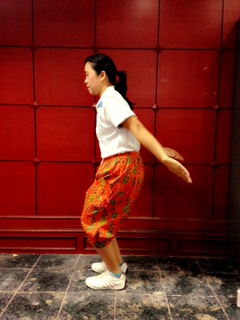

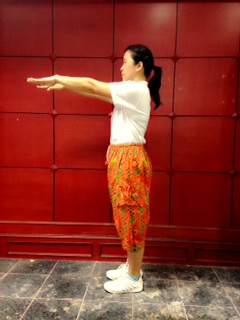


**Instruction:** Subject stand and move both arm in shoulder flexion 90 degree and then move arm down in shoulder extension approximately 15 degree with a little knee flexion.

1. **Ka ten kon tai position**


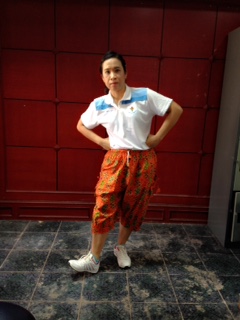

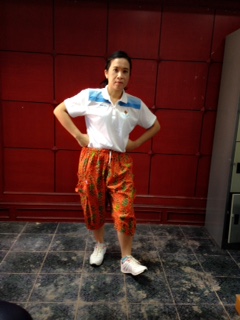


**Instruction:** Subject stand and put both hand on the waist level. Take weight bearing on one leg with a little knee flexion.

1. **Li prai hai hang position**


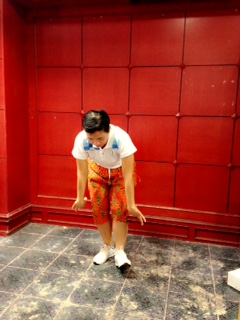

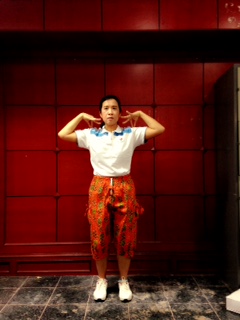

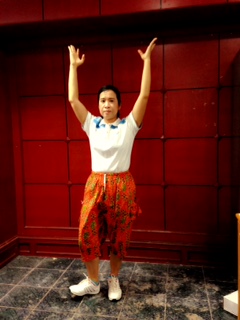


**Instruction:** Subject stand and move both arm in shoulder flexion 0 degree and then move both arm up in shoulder flexion 180 degree with a little knee flexion and take weight bearing on one leg.

1. **Lub mok ka sak position**


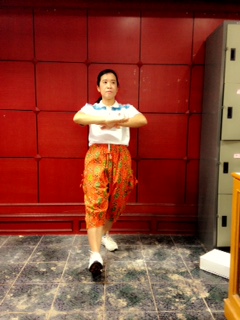

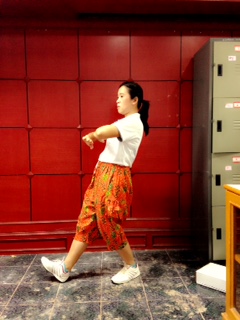

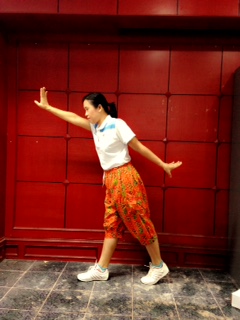


**Instruction:** Subject step forward and lean backward and flexion elbow on chest level and then lean forward and move one arm in shoulder flexion 120 degree and move another arm in shoulder extension 15 degree.

1. **Naw kun sorn position**


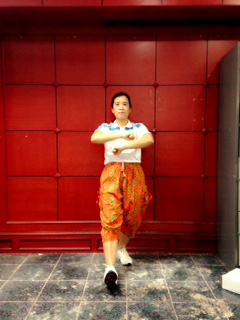

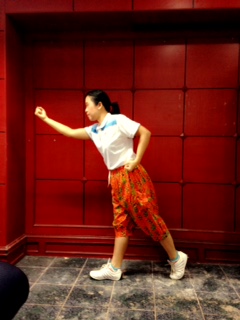


**Instruction:** Subject step forward and lean backward and flexion elbow on chest level with hand clasp and then lean forward and move one arm in shoulder flexion 120 degree and move another arm in shoulder extension 15 degree.

1. **Na kee meun hang position**


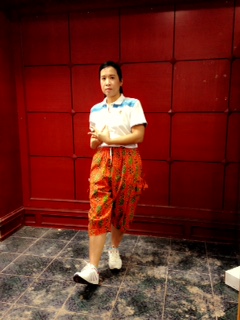

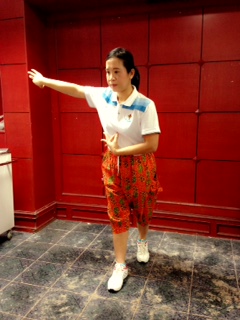

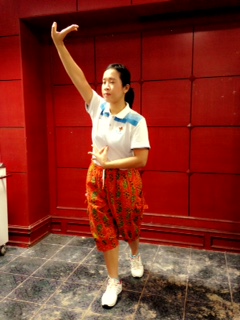

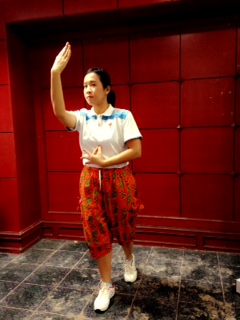


**Instruction:** Subject step forward both arm in elbow flexion on abdomen level with jeab position and lean backward, then move one arm up in shoulder flexion 180 degree and lean forward.

1. **Kwang liao lang position**


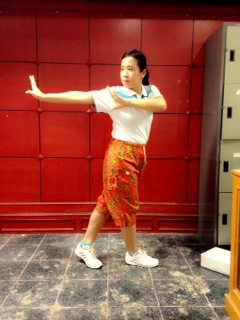

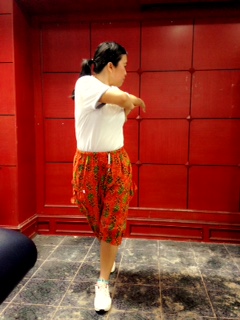


**Instruction:** Subject step forward with twist trunk backward and move arm in shoulder extension 90 degree. Both eyes contact to both hand.

1. **Chang muan nguang position**


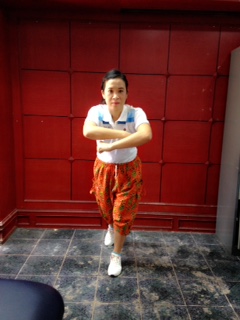

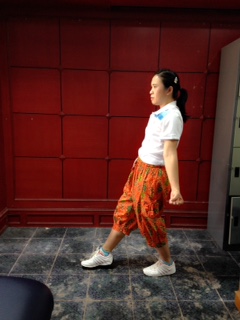


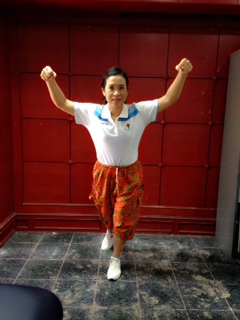

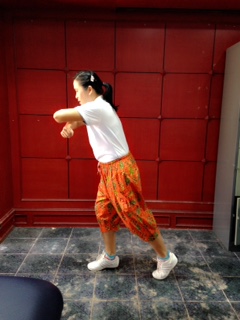


**Instruction:** Subject step forward with lean forward and both arm in elbow flexion 90 degree on chest level, then abduction both arm and lean backward and move one leg forward.

1. **Tuang hug kwang cheu position**


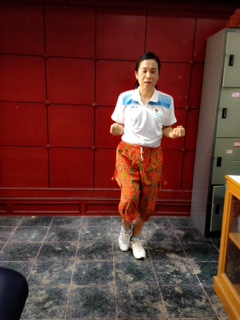

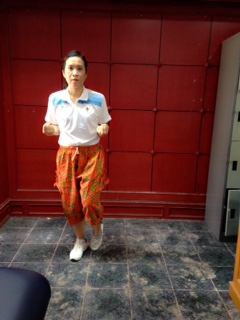

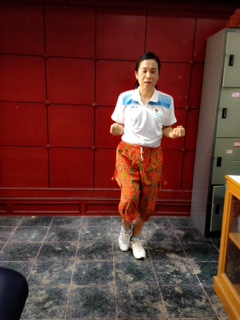

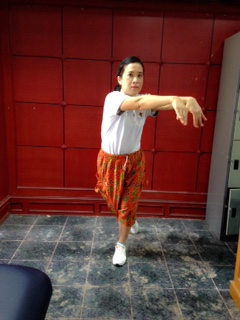


**Instruction:** Subject stand and take weight bearing on one leg and move both arm in elbow flexion 90 degree on waist level and jump 3 time to take weight bearing on one leg and then move arm up in shoulder flexion 90 degree and move one leg back forward.

1. **Yay sam shao position**


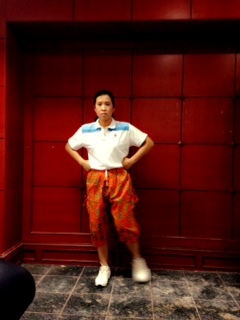

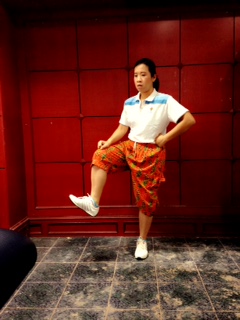

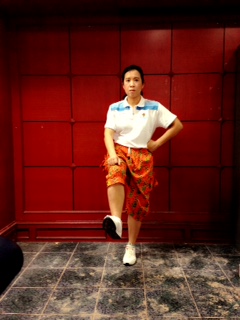


**Instruction:** Subject stand and jump 3 time to take weight bearing on one leg and then lift one leg in hip flexion 90 degree with knee flexion 90 degree.

1. **Lah leab tub position**


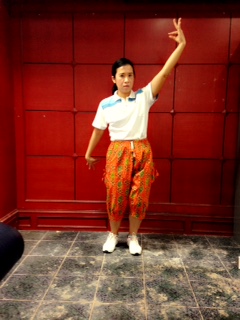

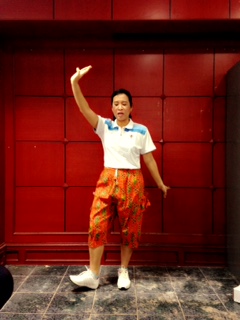


**Instruction:** Subject stand and take weight bearing on one leg and move one arm down in shoulder extension 15 degree and move one arm up in shoulder flexion 180 degree.

1. **Kai leab loa position**


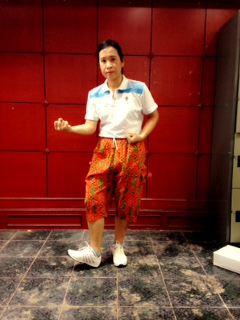

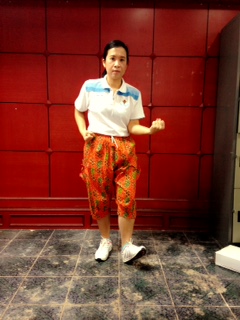


**Instruction:** Subject stand and put both hand on the waist level and move both arm in elbow flexion 90 degree and take weight bearing on one leg with a little knee flexion.

A video of ancient boxing exercise is posted online at <https://goo.gl/ZAdhoU>.
